# COVID-19 Vaccination-Induced Cardiomyopathy Requiring Permanent Left Ventricular Assist Device

**DOI:** 10.7759/cureus.24477

**Published:** 2022-04-25

**Authors:** Muhammad Z Khan, Scott Janus, Sona Franklin, Vincent Figueredo, Abdul Baqi, Rene Alvarez

**Affiliations:** 1 Internal Medicine, St. Mary Medical Center, Langhorne, USA; 2 Cardiology, University Hospitals Cleveland Medical Center, Cleveland, USA; 3 Cardiology, St. Mary Medical Center, Langhorne, USA; 4 Department of Internal Medicine, Mercy St. Vincent Medical Center, Toledo, USA; 5 Heart Failure, Jefferson University Hospital, Philadelphia, USA

**Keywords:** myocarditis, covid-19 vaccination, lvad, covid-19 and cardiomyopathy, covid-19

## Abstract

Myocarditis was identified as a rare but serious adverse event that can occur after mRNA-based coronavirus disease 2019 (COVID-19) vaccination, particularly in young males. During the COVID-19 pandemic, we report a case of a young obese male without multiple comorbidities who presented with abdominal pain and was found to have severe myocarditis/cardiomyopathy, which was likely due to mRNA-based COVID-19 vaccination. The patient had left ventricular assist device (LVAD) implantation during hospitalization. Myocarditis/cardiomyopathy may be a rare complication of the mRNA-based COVID vaccine; however, one should maintain a high index of suspicion that these vaccines may cause irreversible cardiomyopathy if the patient had prior COVID-19 infection.

## Introduction

Since the warp-speed development of the SARS-CoV-2 coronavirus disease 2019 (COVID-19) vaccines (including BNT162b2 (Pfizer-BioNTech), mRNA-1273 (Moderna), and JNJ-78436735 (Johnson & Johnson)), there has been worldwide dissemination of these vaccines to combat the incidence of COVID-19 infection [1]. While COVID-19 vaccine trials demonstrated the vaccines to be overall exceptionally safe, there were documented rare adverse events. Recently, studies have emerged reporting vaccine-associated myocarditis/cardiomyopathy, in particular following mRNA vaccination (BNT162b2 and mRNA-1273) [2]. The incidence of myocarditis following vaccination is incredibly rare, quoted at 10 per 100,000 for the most at-risk group (males 16-30 years old) [3]; however, long-term complications from the resultant myocarditis/cardiomyopathy, especially cardiogenic shock requiring mechanical support, is noteworthy.

In our case report, we describe a remarkable case of COVID-19 vaccination-induced cardiomyopathy with such a profound and permanent left ventricular dysfunction that required durable left ventricular assist device (LVAD) placement.

## Case presentation

A 22-year-old Black male with a past medical history of morbid obesity (body mass index: 45.4 kg/m^2^) and diabetes mellitus contracted COVID-19 in January 2020. He was managed conservatively as an outpatient with no known complications. Six months following the infection, he obtained the COVID-19 (mRNA-1273) initial and booster vaccines (May 19, 2021, and October 7, 2021, respectively). He presented one month after the booster vaccine dose to our community hospital complaining of left lower quadrant abdominal pain. His examination on presentation was significant for a temperature of 36.5°C, pulse rate of 116 beats per minute, respiratory rate of 18 breaths per minute, and blood pressure of 140/84 mmHg. His examination demonstrated a regular rate and tachycardia, with clear lungs. His abdomen was soft and non-tender to palpation without guarding. His blood work showed creatinine of 1.56 mg/dL, INR of 1.2, and troponin of 0.06 mg/dL. His family history was unremarkable for cardiomyopathy or heart disease.

**Figure 1:**
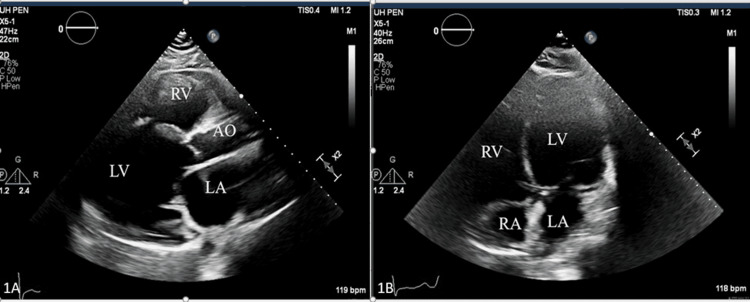
Parasternal long and four-chamber apical view demonstrating severely dilated left ventricle (LV), dilated left atrium (LA), right ventricular (RV) dysfunction, the right atrium (RA), and the aorta (AO).

The patient underwent computed tomography (CT) scan of the abdomen/pelvis, which demonstrated a new infarction of the left kidney. To further evaluate for an embolic source and elevated troponin, a transthoracic echocardiogram (TTE) was obtained, which showed new-onset acute systolic heart failure with a left ventricular ejection fraction (LVEF) of 10% (Figure 1A, 1B).

Due to the concern for a low output state and for further evaluation of the cardiomyopathy, he underwent a coronary angiogram and right heart catheterization. His coronaries were without any significant atherosclerosis; however, his right heart catheterization showed a right atrial pressure of 33 mmHg, pulmonary artery pressure of 70/52 mmHg, wedge pressure of 30 mmHg, cardiac output of 3.11 L/m, and cardiac index of 1.48 L/m/m^2^. He was ultimately transferred to a quaternary care referral center (University Hospitals Cleveland Medical Center) for further evaluation and treatment. He underwent a thorough evaluation for his cardiomyopathy, including thyroid-stimulating hormonal (TSH) (0.72 mIU/L) level, evaluation for infectious source, and metabolic workup, which were all unrevealing.

**Figure 2:**
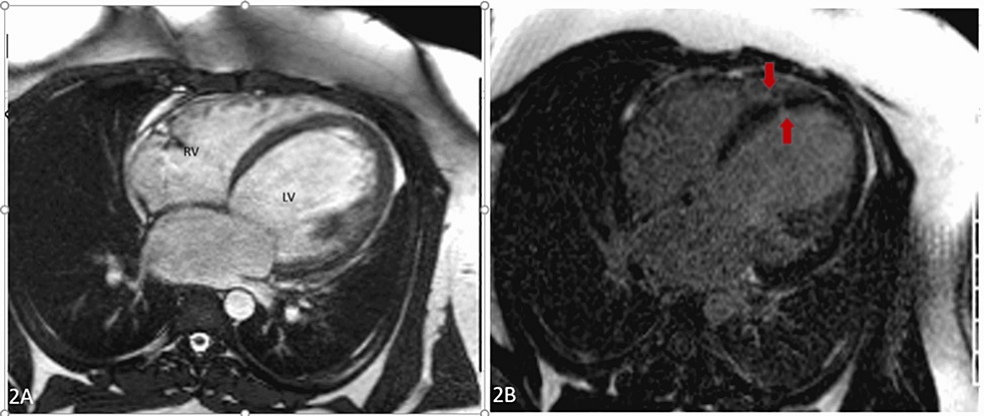
A: Four-chamber view demonstrating markedly dilated right and left ventricle. B: Four-chamber delayed enchantment imaging demonstrating multiple areas of fibrosis. Arrows show embolic infarction. RV: right ventricle, LV: left ventricle

Cardiac magnetic resonance imaging (MRI) was performed, which showed a severely dilated left ventricle (left ventricular end-diastolic volume index (LVEDVI): 195 mL/m^2^) with severely reduced function (quantitative LVEF: 13%). Small focal infarcts were noted in the lateral apical and mid-septal segments, which were likely embolic (Figure 2A, 2B).

**Figure 3:**
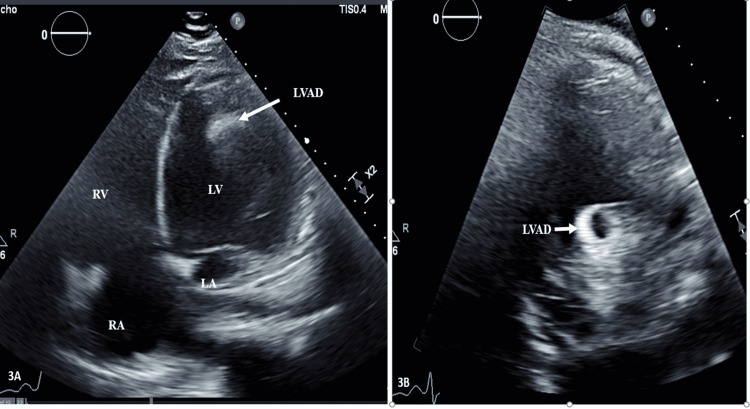
Four-chamber apical view and parasternal long view demonstrating severely dilated left ventricle function with LVAD in place extending from the left ventricle (LV), dilated left atrium (LA), right ventricular (RV) dysfunction, and the right atrium (RA).

No evidence of infiltration, inflammation, or other areas of infarction were noticed on cardiac MRI. His hospital course was complicated by decompensated heart failure and persistent cardiogenic shock initially requiring ionotropic support and ultimately mechanical support with placement of a right axillary Impella device. Despite 43 days of mechanical and ionotropic support, his cardiomyopathy did not improve and required placement of a durable mechanical support device (HeartMate 3 LVAD, Abbott, Chicago, IL, USA) (Figure 3A, 3B).

His postoperative course was complicated by right ventricular dysfunction, which required milrinone, and he was discharged on milrinone. He was discharged to a rehabilitation center with follow-up in the clinic.

## Discussion

To our knowledge, this remarkable case represents the first reported incidence of COVID-19 vaccination-induced cardiomyopathy requiring durable mechanical support. While prior literature noted a wide spectrum of presentations of myocarditis, most patients were asymptomatic and thus likely underrepresented the true incidence [4].

The presentation of myocarditis varies, but most patients are asymptomatic and remain undiagnosed during this period [4]. Post-COVID-19 vaccination, especially after the second dose, and post-COVID-19 infection symptoms are common, with fatigue, fever, and muscle pain being the leading complaints [4]. Some COVID-19 patients might have electrocardiographic (ECG), laboratory, or imaging findings consistent with myocarditis, but further studies are needed. Our patient was asymptotic for some time prior to infarction of the left kidney, which caused abdominal pain.

Myocarditis after vaccination has been reported in several studies. Despite the overall safety and efficacy of the vaccines, there has been a preponderance of myocarditis in the young male population (ages 16-29 years) quoted as high as 10.69 per 100,000 persons [3]. Although more than 2.5 million doses of the vaccine have been delivered and investigated, to our knowledge, only one report of cardiogenic shock requiring temporary mechanical support has been described [3].

The mechanism behind the development of myocarditis is not well understood; however, it is believed to be secondary to molecular mimicry between the spike protein of COVID-19 and the self-antigen that triggers a dysregulation and cascade of immune pathways in response to the mRNA [5]. The presentation of cardiomyopathy after the second vaccine dose suggests that prior exposure to the vaccine was relevant to the hypersensitivity response [5]. In our case, the patient had COVID-19 infection first and was later vaccinated. We postulate that cardiomyopathy was likely a result of the inflammatory process associated with SARS-CoV-2 infection and the subsequent cytokine storm from the vaccination. We also theorize that such a response of the immune system might be elicited in COVID-19-infected patients after vaccination, and it may be the cause of severe myocardial injury requiring mechanical support or heart transplant.

## Conclusions

In this case, we describe that patients who had COVID-19 infection and were later given the mRNA-1273 vaccines may develop irreversible cardiomyopathy, necessitating mechanical cardiovascular support. Also, our case highlights an extreme consequence of dysregulated cytokine/inflammatory storm after the second dose of the COVID-19 vaccine. This case report aimed to shed light on unique sequela from COVID-19 vaccination.
